# Ameliorating the disadvantage for autistic job seekers: An initial evaluation of adapted employment interview questions

**DOI:** 10.1177/1362361320981319

**Published:** 2020-12-18

**Authors:** Katie Maras, Jade Eloise Norris, Jemma Nicholson, Brett Heasman, Anna Remington, Laura Crane

**Affiliations:** 1 University of Bath, UK; 2 University College London, UK

**Keywords:** adaptations, autism, employment, impression management, interviewing, perceptions

## Abstract

Despite possessing valuable skills, social communication differences mean that autistic people are frequently disadvantaged in job interviews. We examined how autistic and non-autistic adults compared on standard (unmodified) job interview questions, and then used these findings to develop and evaluate supportive adaptations to questions. Fifty adults (25 autistic, 25 non-autistic) took part in two mock job interviews. Interview 1 provided a baseline measure of performance when answering typical, unmodified interview questions. Employment experts (unaware of participants’ autism diagnoses) rated all interviewees on question-specific performance and overall impressions and then provided feedback about how interviewees could improve and how questions could be adapted to facilitate this. Interviewees also provided feedback about the interview process from their perspective. Adaptations to the questions were developed, with Interview 2 taking place approximately 6 months later. Results demonstrated that, in Interview 1, employment experts rated autistic interviewees less favourably than nonautistic interviewees. Ratings of both autistic and non-autistic participants’ answers improved in Interview 2, but particularly for autistic interviewees (such that differences between autistic and non-autistic interviewees’ performance reduced in Interview 2). Employers should be aware that adaptations to job interview questions are critical to level the playing field for autistic candidates.

**Lay abstract:**

Despite possessing valuable skills, differences in the way that autistic people understand and respond to others in social situations mean that they are frequently disadvantaged in job interviews. We examined how autistic and non-autistic adults compared on standard (unmodified) job interview questions, and then used these findings to develop and evaluate supportive adaptations to questions. Fifty adults (25 autistic, 25 non-autistic) took part in two mock job interviews. Interview 1 provided a baseline measure of performance when answering typical, unmodified interview questions. Employment experts (unaware of participants’ autism diagnoses) rated all interviewees on their responses to each question and their overall impressions of them and then provided feedback about how interviewees could improve and how questions could be adapted to facilitate this. Interviewees also provided feedback about the interview process, from their perspective. Adaptations to the questions were developed, with Interview 2 taking place approximately 6 months later. Results demonstrated that, in Interview 1, employment experts rated autistic interviewees less favourably than non-autistic interviewees. Ratings of both autistic and non-autistic participants’ answers improved in Interview 2, but particularly for autistic interviewees (such that differences between autistic and non-autistic interviewees’ performance reduced in Interview 2). Employers should be aware that adaptations to job interview questions are critical to level the playing field for autistic candidates.

Despite possessing valuable skill sets, 85% of autistic^1^ people are not in full time work (Knapp et al., 2009) and 46% of the autistic adults who are employed are over-educated or exceed the skill level needed for the roles they are in (Baldwin et al., 2014). More inclusive hiring practices are essential in enabling autistic people to gain access to fulfilling employment. These may range from broad diversity and inclusion plans to actively promoting the employment of people with disabilities (see, for example, Erickson et al., 2014). Indeed, once in work, employers often report that their autistic employees make a valuable contribution to the workplace with their positive personal attributes, skills, and abilities (for reviews, see de Schipper et al., 2016; Scott et al., 2017).

A major barrier to obtaining employment is the initial interview process, which requires social presentation and impression management (IM) skills that autistic people often find challenging (e.g. Chen et al., 2015; Hendrickx, 2008; Higgins et al., 2008; Lorenz et al., 2016; Müller et al., 2003; Richards, 2012; Scott et al., 2019; Strickland et al., 2013). The focus of this study is on specific support and adaptations that employers can make to interview questions to support autistic candidates to provide optimum responses that enable them to compete more equitably with their typically developed peers.

Applicants may employ a range of IM tactics in job interviews to influence interviewer decisions (see Bolino et al., 2008). Theory and evidence suggest that the extent to which an interviewee will engage in IM behaviours is predicted by the interaction between two key factors: (1) an individual’s personality traits and other characteristics, such as engaging in high levels of self-monitoring and being sensitive to the social cues of others (Turnley & Bolino, 2001); and (2) situational factors, such as the structure of the interview (Van Iddekinge et al., 2007). Specifically, IM tactics are more readily employed by those with the characteristics predisposing them to do so, particularly in unstructured and ambiguous situations. However, in more structured situations (where there are uniform expectations to guide behaviour), individuals tend to behave in very similar ways regardless of their individual differences (Tsai et al., 2005; Van Iddekinge et al., 2007).

This is pertinent to autism because traditional theories posit that autistic people experience difficulties in reading others’ intentions and interpreting social cues (see Baron-Cohen, 1997, 2000), which are thought to be underpinned by difficulties in self-monitoring their own internal states (e.g. Grainger et al., 2016; see also Williams, 2010). Recent evidence also indicates that autistic adults are less accurate at predicting how they are perceived by others (Sasson et al., 2018), while others argue that autism is marked by diminished social motivation and reduced concern for reputation management (Cage et al., 2013; Chevallier et al., 2012). Such differences are likely to have significant negative impacts in socially mediated high-stakes contexts such as job interviews.

Typically, employment interviews rely upon open-ended, indirect questions such as ‘Tell me a bit about yourself’ (e.g. Janz, 1982; Levashina et al., 2014). However, difficulties with understanding others’ intentions and inferring what information the employer wants from an answer could be particularly difficult for an autistic person (Baron-Cohen, 1997; Kenworthy et al., 2008; White, 2013; White et al., 2009). For example, being asked to describe a challenge one has experienced in the workplace may not be construed as requiring an answer about how the candidate overcomes adversity or how they proactively address issues that arise. Thus, they may provide a literal response about a time they have encountered a difficulty that does not necessarily present themselves in a favourable way. A further issue is that recalling relevant specific instances from one’s past is often necessary in job interviews to highlight relevant skills and experience (Barrick et al., 2009; Chartered Institute of Personnel and Development, 2018; Levashina et al., 2014), yet autistic people often experience difficulties in recalling specific memories of past experiences, especially at speed (see Crane & Maras, 2018). Together with broad difficulties in executive function (Demetriou et al., 2018), these issues are likely to limit autistic interviewees’ ability to gauge, formulate, and recall a relevant and appropriately detailed response that conveys a positive impression of themselves under standard open-ended questioning (Hendrickx, 2008; Müller et al., 2003).

Critically, it has been theorised that the difficulties experienced by autistic people are most marked on open-ended test situations in which the questions or instructions do not provide the individual with an explicit understanding of the task and what is required of them (White, 2013). These difficulties tend to dissipate in more structured test situations (see Maras, in press). For example, the Task Support Hypothesis (Bowler et al., 1997, 2004) posits that when more support is provided through cued recall and the use of specific prompts, autistic individuals show similar memory recall performance to non-autistic individuals. More structured questioning appears to have a twofold benefit in (1) providing support for executive functions and cognitive processes such as relational processing in memory retrieval while also (2) supporting social cognition by diminishing ambiguity about what is required from their response. More explicit and cued questioning has been shown to be helpful for autistic adults in applied contexts such as the Criminal Justice System (see Maras, in press), but remains to be fully tested in the context of employment interviews (see Norris et al., 2020).

While a recently accumulating body of research has examined how autistic adults can be supported to gain employment, this work has focussed on how the interviewee can be coached to change their behaviours during interviews, neglecting changes that the interviewer can make to questions (e.g. Hillier et al., 2007; Kumazaki et al., 2019; Morgan et al., 2014; Scott et al., 2019; Smith et al., 2014, 2015; Strickland et al., 2013; and see Rashid et al., 2017). While such interventions often have some degree of success, autistic interviewees still frequently provide responses that indicate misunderstanding – for example, with very literal answers (Wehman et al., 2017) or in failing to provide adequate context (Strickland et al., 2013). Recent reconceptualisations of the difficulties experienced by autistic people in understanding others’ thoughts and intentions emphasise communication as a two-way process and that misunderstandings actually reflect a ‘double empathy problem’ (in which both parties misunderstand one another, rather than there being a one-sided ‘impairment’ on the part of the autistic person; Milton, 2012; see also Heasman & Gillespie, 2018; Sheppard et al., 2016). Locating the problem and solution solely within autistic people while disregarding environmental and social barriers is therefore incompatible with theoretical accounts from both autism and IM behaviour perspectives. It also absolves employers of any responsibility to make adaptations and is not conducive to equal participation (Dempsey & Nankervis, 2006; Scott et al., 2019; Shakespeare, 2013).

In sum, successful performance in a job interview requires effective two-way communication between an interviewer and interviewee, in order for questions to be understood in a way that enables the interviewee to formulate an appropriate response that presents themselves favourably. Unless adaptations are made that promote greater shared understanding of what is intrinsically required of an interviewee, autistic candidates are likely to be significantly limited in their ability to emphasise their best attributes and most relevant experience and overshadowed by candidates with a greater predisposition to employ IM tactics. However, employers can make positive adaptations to the interview process, particularly to the questions, which could ameliorate the disadvantage currently experienced by autistic candidates.

The aim of the present study was twofold. First, to gather baseline data regarding how autistic people perform in response to standard (unadapted) employment interview questions compared with non-autistic interviewees (Phase 1). Although interviews have been identified as a major barrier to employment by autistic individuals (e.g. Hendrickx, 2008; Hurlbutt & Chalmers, 2004; Lorenz et al., 2016; Müller et al., 2003) and theoretical accounts of both autism and IM would also predict difficulties, to our knowledge, no research has empirically tested how autistic adults compare with non-autistic adults in employment interviews with standard, unmodified interview questions. The second aim was to use the findings to develop and test adaptations to questions (in Phase 2). Since candidates are often judged not only on the content of their responses but also on interviewers’ overall perceptions of them (Barrick et al., 2009), employers rated the quality of interviewees’ responses to each question individually, as well as rating their overall impressions of the candidates across the entire interview. In accordance with the growing recognition of the need to include autistic perspectives (Chamak et al., 2008; see also Pellicano et al., 2018), feedback was also sought from interviewees regarding their perceptions of the interview questions and aspects of the interview they found challenging or supportive.

We predicted that differences in social communication, IM, executive functioning, and memory would result in autistic interviewees being perceived less favourably by employer raters than non-autistic interviewees in response to standard, open-ended, interview questions (in Phase 1). In line with the Task Support Hypothesis, we predicted that when more structured, explicit, and supportive questions were used (in Phase 2), their responses would be improved to the extent that the difference between groups would be ameliorated.

## Method

### Participants

#### Interviewees

A power analysis using G*Power3.1 (Faul et al., 2007) indicated that a sample size of 40 would give 80% power to detect a medium-to-large effect of group and interview question adaptations (i.e. to have significant implications for practice). A total of 50 participants took part in Phase 1 of the study: 25 autistic (15 males, 10 females) and 25 non-autistic (5 males, 20 females). Of these, 21 autistic (12 males, 9 females) and 21 non-autistic (5 males, 16 females) returned to complete the second interview in Phase 2. Autistic and non-autistic participants were recruited primarily from the Centre for Applied Autism Research (CAAR) database at the University of Bath and through ongoing recruitment, including via social media, support groups, and the local community (posters, magazine articles, etc.) across the South West of England. All autistic participants had received a formal clinical diagnosis of Autism Spectrum Disorder according to *Diagnostic and Statistical Manual of Mental Disorders* (4th ed.; DSM-IV; American Psychiatric Association, 2000) or *Diagnostic and Statistical Manual of Mental Disorders* (5th ed.; DSM-5; American Psychiatric Association, 2013) criteria, which was confirmed with a copy of their diagnostic report. Those who had received a diagnosis but were unable to access their report received the Autism Diagnostic Observation Schedule–Second Edition (ADOS-2; Lord et al., 2012) to confirm the diagnosis.

Autistic and non-autistic groups did not significantly differ on age, *t*(48) < 0.01, *p* = 1.00, *d* < 0.01, or on measures from the Wechsler Abbreviated Scale of Intelligence–Second Edition (WASI-II; Wechsler, 2011): Verbal Comprehension Index (VCI), *t*(48) = 0.47, *p* = 0.644, *d* = 0.13; Perceptual Reasoning Index (PRI), *t*(48) = 1.07, *p* = 0.289, *d* = 0.30; or Full Scale Intelligence Quotient (FSIQ), *t*(42.87) = 0.10, *p* = 0.325, *d* = 0.28. All non-autistic participants scored below the recommended minimum cut-off of 26 on the Autism Spectrum Quotient (AQ-50), which measures levels of autistic traits (Baron-Cohen et al., 2001; see Woodbury-Smith et al., 2005). As expected, the autistic group’s AQ scores were significantly higher than those of the non-autistic group, *t*(40.10) = 10.29, *p* < 0.001, *d* = 2.95 (Table 1).

**Table 1.** Age, WASI-II, and AQ scores by group (standard deviations are in parentheses).

	Phase 1		Phase 2	
	Autistic adults (*n* = 25)	Non-autistic adults (*n* = 25)	Autistic adults (*n* = 21)	Non-autistic adults (*n* = 21)
Age (years)	34.24 (12.95); range = 18–59	34.24 (12.21); range = 18–60	35.81 (13.27); range = 18–59	32.71 (10.75); range = 18–51
VCI	107.12 (10.81); range = 85–128	108.44 (9.22); range = 79–125	106.33 (10.61); range = 85–128	108.33 (9.18); range = 79–119
PRI	106.84 (14.32); range = 82–131	110.76 (11.34); range = 92–136	107.14 (14.47); range = 82–131	109.38 (11.31); range = 92–136
FSIQ	107.88 (12.52); range = 89–132	110.92 (8.73); range = 88–124	107.57 (12.73); range = 89–132	110.10 (8.75); range = 88–123
AQ-50	34.33 (9.16); range = 14–46	11.28 (6.17); range = 2–24	35.80 (7.63); range = 20–46	11.43 (6.62); range = 2–24

WASI-II: Wechsler Abbreviated Scale of Intelligence–Second Edition; VCI: Verbal Comprehension Index; PRI: Perceptual Reasoning Index; FSIQ: Full Scale Intelligence Quotient; AQ-50: Autism Spectrum Quotient.

To better characterise our sample, information was collected on participants’ current education, work status, and their highest level of educational attainment. More non-autistic interviewees were in full-time work; otherwise, both groups were comparable in terms of current education/employment status and were similarly educated to a high level (Table 2).

**Table 2.** Autistic and non-autistic interviewees’ highest level of educational attainment and current education/employment status.

	% autistic participants (*n*)	% non-autistic participants (*n*)
Previous work experience	87.5 (21)	96 (24)
Current work/education status (categories not mutually exclusive)		
Full-time work	4.2 (1)	44 (11)
Part-time work	20.8 (5)	24 (6)
Full-time education	29.2 (7)	28 (7)
Part-time education	8.3 (2)	8 (2)
Volunteering	12.5 (3)	8 (2)
Not working, looking for work	4.2 (1)	4 (1)
Not working, not looking for work	33.3 (8)	12 (3)
Yes, self-employed	12.5 (3)	8 (2)
Full-time carer	8.3 (2)	0 (0)
Off sick	4.2 (1)	0 (0)
Highest level of educational attainment		
Master’s level or above	16 (4)	4 (1)
Undergraduate degree	41.7 (10)	44 (11)
A level or equivalent (typically at age 16–18)	28 (7)	48 (12)
GCSEs or equivalent (typically at age 14–16)	12.5 (3)	0 (0)
Other	0 (0)	4 (1)

GCSE: General Certificate of Secondary Education.

#### Employer raters

Four independent employment professionals (three females, one male) were recruited via the researchers’ professional contacts (within the employment industry) to rate transcripts of participants’ answers. The raters worked in various roles for different companies (pharmaceuticals, banking, manufacturing, and strategic intelligence) and all had substantial experience in recruiting and interviewing. Their ages ranged from 38 to 52 (*M* = 45, *SD* = 5.77) years. To provide an index of their knowledge, experience, and perceptions of autism, employers also completed brief scales. These demonstrated scores within the average range to those previously reported: Autism Awareness Scale (measuring knowledge of autism; Gillespie-Lynch et al., 2015): *M* = 9.75, *SD* = 5.06, range = 4–16; Level of Contact Scale (measuring personal experience of autism; Morrison et al., 2019): *M* = 5.25, *SD* = 2.87, range = 3–9; Social Distance Scale (measuring stigma against autistic people; Gillespie-Lynch et al., 2015): *M* = 7.75, *SD* = 2.06, range = 6–10.

#### Ethical considerations

Participants provided informed written consent to take part in the study and were fully debriefed. Ethical approval was obtained from the Psychology Research Ethics Committee at the University of Bath.

### Design

The study utilised a 2 (Group: autistic vs non-autistic) × 2 (Phase: Phase 1 unadapted questions vs Phase 2 adapted questions) mixed design, where Phase was within participants. In Phase 1, all participants answered standard employment interview questions from one of two interview schedules (A or B). In Phase 2, the same participants returned to answer adapted interview questions from the interview schedule they did not receive at Phase 1. Dependent variables were employment professionals’ quantitative scale ratings of interviewees’ answers to each of the seven interview questions and their overall impression of the interviewee measured on nine aspects of participants’ overall performance (see below for details). Qualitative feedback was also obtained from both employers and interviewees to inform the development of question adaptations for Phase 2 and provide a more in-depth exploration of the efficacy of the different question types.

### Materials

#### Interview schedules

Two interview schedules (A and B) were developed, each with seven questions typical of standard employment interviews. Questions included those aimed at eliciting descriptions of experience and activity, personality characteristics, and self-evaluative information (e.g. ‘What are some of your strengths?’), as well as past job experience and situational judgements (e.g. ‘Tell me about a time you had to work with someone who was difficult to get along with – how did/would you handle it?’; Campion et al., 1997; Janz, 1982; Salgado & Moscoso, 2002). Schedules A and B comprised different questions to avoid practice effects between Phases 1 and 2, but were each balanced with parallel questions aimed at eliciting descriptions of previous experience, descriptions of past behaviour, and self-evaluation (see Supplemental Appendix A).

#### Employer ratings of interviewees’ responses

##### Content of responses

Employment professionals’ ratings of the quality of interviewees’ responses to each question were scored using an adapted form of the Interview Skills Rating Instrument (Strickland et al., 2013). The original scale was adapted from a 4- to a 5-point scale, ranging from 1 (*very poor*) to 5 (*excellent*) to provide a more fine-grained distinction between those who barely answered the question at all (e.g. with a single yes/no response, to receive a score of 1) and those who did respond, but poorly (e.g. with one or two sentences, to receive a score of 2). To inform the development of adaptations to questions for Phase 2, employers were also asked two optional open-ended questions relating to each interviewee’s response to each interview question in Phase 1: ‘How could the interviewee improve their answer?’ and ‘How could the question be adapted to support this?’ (see Supplemental Appendix B for employer rating questions and scoring criteria).

##### Overall impressions

Employers’ ratings of their overall impressions of each interviewee were obtained after they had finished rating each interview on nine aspects of interviewees’ general performance: confidence, motivation, knowledgeability, conscientiousness, competence, intelligence, likeability, communication skills, and how easy they would be to work with, rated on a 5-point Likert-type scale ranging from *not at all* to *extremely*. These constructs were identified in a review by Huffcutt (2011) as factors on which employers base their interview ratings^2^ (see also Salgado & Moscoso, 2002; Smith et al., 2014).

#### Interviewee feedback survey

At the end of each interview, interviewees completed an online Qualtrics survey about their experience of the interview. This included questions about their confidence in their performance and how clear the desired/required responses were from the questions (on a 5-point Likert-type scale ranging from *not at all* to *extremely*), as well as which questions they found easy/challenging and why, and what would have made the interview easier for them. In addition, at Phase 2, interviewees were also asked whether they used the printout of the questions (details below) and how useful they found this, as well as how this second interview compared with the interview that they had received in Phase 1.

### Procedure

#### Phase 1

Participants completed the mock employment interview in a quiet room at the University of Bath. All participants were interviewed by the second author (J.E.N.) in the same room. Participants were not provided with a specific job description (for parity across participants with different levels of skills and experience), but were asked to answer the questions as though they were in a real employment interview. Participants were pseudo-randomly allocated to receive either schedule A or B at Phase 1, within the constraints of ensuring groups were matched on age and intelligence quotient (IQ) (all *p*s > 0.760). Interviewees were asked each of the seven interview questions in the same order. In cases where participants could not provide an answer, they were given plenty of time, but the interviewer moved on to the next question if they were still unable to answer (they could not return to the unanswered question).^3^ The interviewer provided minimal verbal feedback throughout, but the question was repeated or clarified if requested (limited to rephrasing the question or using synonyms). After the interview, participants completed the online feedback survey asking them about their experience of the interview.

#### Phase 2

Participants returned around 6 months later (*M* = 27.2 weeks, *SD* = 3.89, range = 16–32 weeks) to receive adapted questions from the alternative interview schedule to that completed at Phase 1. Again, they were told that there was no specific job description and that they should answer the questions as though in a real employment interview. The interviews were conducted in the same way as at Phase 1 (following feedback from employers and interviewees; see the “Phase 2a: Development stage” subsection of the “Results” section for details), with three exceptions. First, to negate any potential increases in executive demands due to the multi-part nature of the adapted questions, participants were asked to respond to each part of the question in turn. Second, and relatedly, they were provided with a printout of the questions, which remained in front of them throughout the interview. Third, interviewees were given more explicit instructions regarding the structure of the interview, such as the number of questions they were going to be asked and when they should provide an answer. At the end of the interview, participants repeated the online feedback survey about their experience of the interview.

#### Employer ratings

After each phase of testing, the employer raters received anonymised and group-blinded transcripts of the interviews alongside a document providing operational guidance on how to provide the ratings. They were informed that participants were completing a mock employment interview as part of a psychological study. For Phase 2, employers were asked to rate participants’ answers independently of their ratings in Phase 1 (should they have remembered these).

### Analysis plan

Results are reported by phase. For Phase 1 (when standard questions were used), between-group comparisons examined whether employers’ quantitative ratings differed between autistic and non-autistic interviewees in terms of (1) question-specific performance and (2) their overall impressions of them. Phase 2 is presented in two parts. First, a development stage is reported in which a content analysis of employers’ qualitative feedback from Phase 1 about how interviewees’ performance could be improved and how questions could be adapted to support this (conducted by J.E.N. and J.N.) and interviewees’ reflections on their Phase 1 interview experiences (conducted by B.H.) was used to guide the development of adaptations to questions for Phase 2. This involved an initial inductive content analysis (Mayring, 2015), whereby the texts were coded and systematically classified into themes of similar meaning, before the salience of themes was interpreted through the quantitative count of codes (Vaismoradi et al., 2013). Potential adaptations to questions were developed based on the themes identified from employers’ and interviewees’ feedback. K.M., J.E.N., and J.N. initially identified potential adaptations and these were then refined in consultation with the other authors, six autistic adults, and a specialist autism employment support and training professional (from www.asmentoring.co.uk).

Phase 2 interview data (evaluating the effect of the interview question adaptations) were analysed using Group × Phase mixed models. These tested the effects of the adapted questions on employers’ ratings of autistic versus non-autistic interviewees’ performance and their overall impressions of them. A content analysis (conducted by B.H.) then examined how the adapted interview questions affected the interview experience as reported by autistic and non-autistic interviewees, while mixed analyses of variance (ANOVAs) tested whether the adapted questions improved autistic and non-autistic interviewees’ ratings of confidence in their performance and how clear they felt the desired responses were from the questions.

## Results

### Preliminary analyses

Inspection of employer ratings data revealed an outlier in the autistic group who received unusually low rating scores across all measures (>2.5 *SD*s below the mean). This participant was removed from the subsequent quantitative analyses of employers’ ratings (NB. the pattern of findings remained the same). Interview schedule (A or B) had no effect on employer ratings of interviewees’ responses to questions or on their overall impression ratings in either Phase 1 or Phase 2 (all *p*s > 0.403). Subsequent analyses were therefore collapsed across interview schedule.

### Phase 1: unadapted questions

#### Quality and content of interviewees’ responses

**Figure 1.**
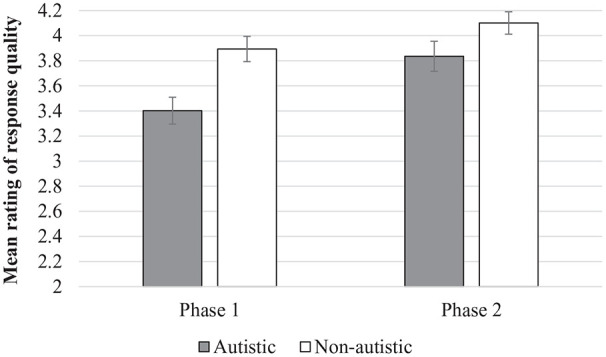
Employers’ mean ratings of autistic and non-autistic interviewees’ responses to questions at Phase 1 and Phase 2 (error bars represent standard errors of the mean).

An independent-samples *t* test indicated that employers’ mean ratings of autistic interviewees’ responses across the seven unadapted questions^4^ in Phase 1 were significantly lower (*M* *=* 3.41, *SD* *=* 0.46) than their ratings of non-autistic interviewees’ responses (*M* *=* 3.91, *SD* *=* 0.50), *t*(47) = 3.61, *p* *=* 0.001, *d* *=* 1.03 (Figure 1).

#### Employers’ overall impressions

A multivariate analysis of variance (MANOVA), with Group as the independent variable and mean ratings on each of the nine impressions items as the dependent variables, indicated a significant multivariate effect of Group on employers’ overall impressions of interviewees in Phase 1, Pillai’s Trace = 0.50, *F*(9, 39) = 4.32, *p* = 0.001, $\eta_{p}^{2}$ = 0.50. Separate univariate tests on the outcome variables revealed significant effects of Group (with autistic interviewees being rated more poorly) in terms of confidence, communication skills, likeability, and ease to work with (all *F*s(1, 47) > 7.58, *p*s < 0.008, $\eta_{p}^{2}$s > 0.14) (see Table 7). Groups did not significantly differ on employers’ overall impressions of their motivation, knowledge, conscientiousness, competence, or intelligence (all *F*s(1, 47) < 3.64, *p*s > 0.063, $\eta_{p}^{2}$s < 0.07) (Table 7).

### Phase 2a: development stage

#### Employers’ feedback

##### How could participants could improve their answers?

The major theme categories that were identified from the content analysis of employers’ suggestions for how interviewees could improve their answers fell under (1) *Content: General*, (2) *Content: Self-reflection*, (3) *Focus*, (4) *Length*, and (5) *Delivery* (see Table 3). In summary, employers most frequently suggested that participants in both groups – but particularly autistic interviewees – could improve the *content* of their responses with more effective use of examples, providing more specific detail and explaining how situation outcomes were achieved (e.g. what steps they took to achieve this). They also suggested that interviewees should show more positive *self-reflection* in their responses by reducing negative comments made about themselves and highlighting what they learnt from situations. Employers also recommended that autistic (and to a lesser extent non-autistic) interviewees needed to *focus*, think before responding, maintain clarity, and directly answer the question. Many answers provided by both autistic and non-autistic interviewees were criticised for lacking structure and for inappropriate *length* (too short/too long). Employers also suggested that both autistic and non-autistic interviewees’ *delivery* needed to be more positive/confident and that autistic interviewees’ responses in particular would be improved if they were less hesitant.

**Table 3.** Codes identified under each theme from the content analysis of employer feedback regarding how interviewees’ answers to questions could be improved.

	Autistic	Non-autistic
Content: General		
Use examples (effectively)	130	90
Give more relevant specific detail	125	60
Explain management strategies	21	23
Explain how the end result was achieved	25	15
Describe result/outcomes	11	7
Give less irrelevant detail	7	2
Explain why	4	2
Use better examples	6	5
Content: Self-reflection		
Reduce negative comments about the self	34	15
Describe learning	16	13
Focus on self/own role	2	7
Describe enjoyment	7	8
Describe emotional impact/experience	6	1
Demonstrate skills	2	5
Focus		
Think before answering/focus/clarity on question	114	84
Answer the question	21	11
Avoid over-literal answer	6	3
Length		
Answer too long	45	43
Answer too short	24	18
Delivery		
Be more confident/positive/enthusiastic	50	42
Structure answer better	20	25
Less hesitant	19	11

##### How could the questions be adapted?

Main themes for employers’ suggested adaptations were identified for each question from schedules A and B (i.e. 14 questions in total). Codes broadly fell under one of four over-arching themes (Table 4). Overall, these themes closely reflected their comments about how interviewees could improve their answers, as described above. Indeed, the most common themes included that the interviewer should *prompt for specific information* and *self-reflection* (e.g. what the interviewee learned from a situation). Employers also frequently suggested that interviewees should be given more *guidance on how to respond* and how the *question structure* could be adapted to facilitate this (e.g. by asking questions in multiple parts).

**Table 4.** Codes identified under each theme from the content analysis of employer feedback regarding how the questions could be adapted for autistic and non-autistic interviewees.

	Autistic	Non-autistic
Prompt for specific information		
Ask for examples	138	107
Ask for details	73	46
Ask what the outcome was	59	25
Ask about management strategies	37	40
Ask how (what actions were/ would be taken)	13	7
Request work history	9	5
Ask why	6	3
Ask for application in work	4	5
Ask about skills used	2	1
Prompt for self-reflection		
Ask about learning/skills gained	28	19
Prompt to refer to self/focus on own role in situation	28	13
Ask about benefits/enjoyment	13	7
Ask about aspirations/goals	6	5
Ask for feelings	6	4
Guidance on how to respond		
Specify how to structure response	69	41
Provide guidance on timeframe	14	15
Provide guidance on what to prioritise in answer	3	3
Request short response	4	5
Question structure		
Split question/ask follow-ups	19	18

#### Interviewees’ feedback

The content analysis resulted in four organising themes: (1) *self-reflections*, (2) *interpersonal dynamic*, (3) *question clarity and structure*, and (4) *adaptations*. Table 5 presents the themes, subthemes, and their coding frequency for autistic and non-autistic participants. Interviewees *self-reflected* on aspects of their performance relating to cognition, emotion, and communication. The autistic group reported more cognitive difficulties (e.g. processing the question asked or recalling the appropriate memory) and slightly higher communication challenges (e.g. question–answer fit, staying on topic, verbal and non-verbal articulation) compared with the non-autistic group. Within the *interpersonal dynamic* of the interview, both groups reported using strategies to manage impressions, but the non-autistic group were more likely to report concerns about how to ‘sell’ themselves, downplaying their difficulties and boosting their strengths, while the autistic group expressed concerns about being too honest.

**Table 5.** Interviewees’ reflections on their interview experiences at Phase 1 (unadapted questions).

Themes	Autistic	Non-autistic
Self-reflections		
Cognition (positive/negative)	35/42	62/29
Emotion (positive/negative)	9/23	13/22
Communication (positive/negative)	16/21	19/15
Interpersonal dynamic		
Using strategies for managing impressions	19	23
Interpreting interviewer aims (easy/difficult)	2/10	2/14
Question clarity structure		
Questions unclear and structured poorly	40	29
Questions clear and structured well	29	36
Adaptations		
More specific questions	20	16
Visual supports	21	4
Questions that allow for more flexible answers	11	9
More feedback on questions	7	9
More time to prepare and respond	6	2

Reflecting on *question clarity and structure*, autistic interviewees were more likely to report that questions were unclear and poorly structured than the non-autistic group. Accordingly, both autistic and non-autistic interviewees suggested that *adaptations* should include the use of more specific questions. Autistic interviewees felt that visual supports (e.g. a CV printout, a question printout, taking prepared notes to the interview) would be particularly helpful. Both groups also reported a wish for questions with more flexible answers (e.g. that enabled them to draw more widely on their experiences), more feedback on questions, and having more time to prepare and respond to questions.

#### Adaptations to questions

Questions were adapted based on the qualitative analyses of employer and interviewee feedback, primarily aiming to make the desired response more explicit, for example, by requesting more specific information and details and supporting the interviewee to structure their answer clearly with effective use of examples. This was achieved by adapting the question wording such that the interviewee was first oriented to the topic (to set the context), before breaking the question down into separate, specific questions (to request particular details while avoiding compound questions with multiple and/or stacked clauses). Table 6 provides examples (see Supplemental Appendix A for the full list of adapted questions). All interviewees were also provided with printouts of the adapted questions in Phase 2, which were visible throughout the interview to further support their comprehension of questions, in line with previous suggestions by both employers and interviewees.^5^

**Table 6.** Examples of question adaptations.

Phase 1 (unadapted)	Phase 2 (adapted)
What are some of your strengths?	I’m going to ask about your strengths: • What do you consider to be your main strengths (things that you are good at)? • How have you used these strengths at work [in education]?
What experience do you have of managing high workloads?	Think of an example of when you’ve had lots of tasks to complete in a limited amount of time. Please tell me: • What was the situation? • What management strategies did you use? • Were these strategies effective?
Tell me about a time you’ve disagreed with a colleague – how did/would you handle it?	Think of a time you’ve disagree with a colleague. Please tell me: • What was the disagreement about? • What *you* did to resolve it?

### Phase 2b: effectiveness of adaptations

#### Employers’ quantitative ratings of interviewees in Phase 1 versus Phase 2

##### Quality and content of interviewees’ responses

A 2 (Group) × 2 (Phase) mixed ANOVA was used to examine whether the question adaptations improved the quality of autistic and non-autistic interviewees’ responses (the dependent variable was employers’ mean ratings of interviewees’ responses to the seven questions within each phase). There was a significant main effect of Group, *F*(1, 39) = 7.37, *p* = 0.010, $\eta_{p}^{2}$ = 0.16, whereby autistic interviewees’ responses were rated less favourably (*M* *=* 3.62, *SD* *=* 0.44) than non-autistic interviewees’ responses overall (*M* *=* 4.00, *SD* *=* 0.45). There was also a main effect of Phase, *F*(1, 39) = 39.65, *p* < 0.001, $\eta_{p}^{2}$ = 0.50, with employers rating all interviewees’ responses to the adapted questions in Phase 2 more positively (*M* = 3.97, *SD* *=* 0.44) than at Phase 1 (*M* *=* 3.65, *SD* *=* 0.57). Crucially, there was a Group × Phase interaction, *F*(1, 39) = 5.03, *p* *=* 0.031, $\eta_{p}^{2}$ = 0.11. As can be seen in Figure 1, while autistic interviewees’ responses were rated less favourably than non-autistic interviewees at Phase 1 (*p* *=* 0.010, *d* *=* 0.85), the difference between groups at Phase 2 was not significant (*p* *=* 0.055, *d* *=* 0.62).

##### Employers’ overall impressions

A 2 (Group) × 2 (Phase) mixed MANOVA examined whether employers’ overall impressions of interviewees on each of the nine items improved following the adaptations to questions. There were significant multivariate effects of Group, Pillai’s Trace = 0.55, *F*(9, 31) = 4.13, *p* = 0.001, $\eta_{p}^{2}$ = 0.55, and Phase, Pillai’s Trace = 0.51, *F*(9, 31) = 3.63, *p* *=* 0.003, $\eta_{p}^{2}$ = 0.51, but no Group × Phase interaction, Pillai’s Trace = 0.27, *F*(9, 31) = 1.25, *p* *=* 0.303, $\eta_{p}^{2}$ = 0.27.

Separate univariate tests on the outcome variables revealed significant effects of Group (with autistic interviewees again being rated less favourably) in terms of confidence, communication skills, likeability, and perceived ease to work with (all *F*s(1, 39) > 4.54, *p*s < 0.039, $\eta_{p}^{2}$s > 0.10). Groups did not significantly differ on employers’ overall impressions of their motivation, knowledge, conscientiousness, competence, or intelligence (all *F*s(1, 39) < 2.87, *p*s > 0.098, $\eta_{p}^{2}$s < 0.07) (Table 7).

**Table 7.** Employer ratings of overall impressions of autistic and non-autistic interviewees in Phases 1 and 2 (standard deviations are in parentheses).

Overall impressions	Phase 1 (unadapted)					Phase 2 (adapted)					Phase 1 vs Phase 2		
	Autistic	Non-autistic	*F*	*p*	$\eta_{p}^{2}$	Autistic	Non-autistic	*F*	*p*	$\eta_{p}^{2}$	*F*	*p*	$\eta_{p}^{2}$
Confidence^a^	1.92 (0.66)	2.47 (0.73)	7.58	0.008	0.14	2.13 (0.64)	2.48 (0.57)	4.54	0.039	0.10	1.24	0.273	0.03
Motivation	2.31 (0.62)	2.63 (0.65)	3.03	0.088	0.06	2.43 (0.59)	2.74 (0.59)	2.87	0.098	0.07	1.69	0.202	0.04
Knowledge	2.32 (0.54)	2.59 (0.61)	2.55	0.117	0.05	2.53 (0.55)	2.51 (0.52)	0.36	0.550	0.01	1.15	0.291	0.03
Conscientiousness^b^	2.52 (0.48)	2.72 (0.53)	1.80	0.186	0.04	2.81 (0.43)	2.98 (0.37)	1.32	0.257	0.03	12.62	0.001	0.24
Competence^b^	2.39 (0.54)	2.71 (0.61)	3.64	0.063	0.07	2.63 (0.50)	2.77 (0.46)	1.65	0.206	0.04	8.73	0.005	0.18
Intelligence	2.34 (0.57)	2.50 (0.62)	0.96	0.333	0.02	2.45 (0.45)	2.57 (0.40)	0.45	0.508	0.01	1.39	0.246	0.03
Communication skills^a,b^	1.93 (0.57)	2.59 (0.66)	14.14	<0.001	0.23	2.28 (0.62)	2.86 (0.52)	11.95	<0.001	0.24	18.65	<0.001	0.32
Likeability^a,b^	2.33 (0.50)	2.75 (0.46	9.14	0.004	0.16	2.56 (0.44)	2.83 (0.35)	9.00	0.005	0.19	4.21	0.047	0.10
Ease to work with^a,b^	2.08 (0.55)	2.75 (0.43)	22.56	<0.001	0.32	2.38 (0.53)	2.80 (0.40)	15.84	<0.001	0.29	7.10	0.011	0.15

a Significant main effect of Group (across phases). ^b^Significant main effect of Phase (across groups).

Univariate tests on the outcome variables revealed that employers’ overall impressions of interviewees’ conscientiousness, competence, communication skills, likeability, and ease to work with all improved with the provision of adaptations to the questions in Phase 2 (all *F*s(1, 39) > 4.21, *p*s < 0.047, $\eta_{p}^{2}$s > 0.10). There were no effects of Phase on impressions of interviewees’ confidence, motivation, knowledge, or intelligence (all *F*s(1, 39) < 1.69, *p*s > 0.202, $\eta_{p}^{2}$s < 0.04) (Table 7).

#### Interviewees’ feedback of interviews at Phase 2

Interviewees’ reflections on their interview experience in Phase 2 were coded by B.H. using the content analysis process as outlined for Phase 1. Table 8 presents the themes, subthemes, and their coding frequency for autistic and non-autistic participants. Key differences at Phase 2 (compared with Phase 1) included a diminution in reported cognitive and communication difficulties and a notable reduction in reports of struggling to interpret the interviewer aims by both groups. There was also a reduction in the perception that questions were unclear for both autistic and non-autistic groups and both groups reported fewer observations that more feedback was needed for questions in Phase 2, suggesting the adaptations had a pervasively positive effect. Reflections on the adaptations showed that the printouts of the questions were positively received by both autistic and non-autistic interviewees, with participants remarking that it helped to structure their responses, stay focussed on the question in hand, and to understand where the interview was heading so that they could avoid answering future questions too early. Nevertheless, a small number of interviewees also reported drawbacks, such as finding the printout a distraction from looking at the interviewer or disrupting the natural flow of dialogue.

**Table 8.** Interviewees’ reflections on their interview experiences at Phase 2 (adapted questions).

Themes	Autistic	Non-autistic
Self-reflections		
Cognition (positive/negative)	13/15	33/16
Emotion (positive/negative)	2/14	6/6
Communication (positive/negative)	10/8	16/8
Interpersonal dynamic		
Using strategies for managing impressions	15	15
Difficulty interpreting interviewer aims	2	3
Question clarity structure		
Questions unclear and structured poorly	37	26
Questions clear and structured well	2	6
Reflections on current adaptations		
Print out of questions helpful	29	38
Print out of questions unhelpful	5	4
Suggestions for further adaptations		
More specific questions	7	3
Questions that allow for more flexible answers	6	3
More feedback on questions	0	1

Finally, a 2 (Group) × 2 (Phase) mixed ANOVA on interviewees’ ratings of their confidence in their performance indicated a main effect of Phase, *F*(1, 40) = 4.12, *p* = 0.049, $\eta_{p}^{2}$ = 0.09, with interviewees reporting significantly higher confidence at Phase 2 (*M* *=* 3.55, *SD* = 0.77) compared with Phase 1 (*M* *=* 3.24, *SD* = 1.14). There was no main effect of Group, *F*(1, 40) = 3.23, *p* = 0.080, $\eta_{p}^{2}$ = 0.08, or Group × Phase interaction for interviewees’ confidence ratings, *F*(1, 40) = 1.98, *p* = 0.168, $\eta_{p}^{2}$ = 0.05. A Group × Phase ANOVA on interviewees’ ratings regarding the clarity of questions again indicated a main effect of Phase, *F*(1, 40) = 17.68, *p* < 0.001, $\eta_{p}^{2}$ = 0.31, with interviewees reporting that questions were clearer at Phase 2 (*M* *=* 4.40, *SD* = 0.59) compared with Phase 1 (*M* = 3.69, *SD* = 1.09). There was no main effect of Group, *F*(1, 40) = 2.79, *p* = 0.103, $\eta_{p}^{2}$ = 0.07, but there was a Phase × Group interaction for question clarity ratings, *F*(1, 40) = 5.03, *p* = 0.031, $\eta_{p}^{2}$ = 0.11. Specifically, while the autistic group reported significantly lower question clarity (*M* = 3.31, *SD* = 0.74) than non-autistic participants (*M* = 4.08, *SD* = 1.16) at Phase 1 (*p* = 0.009), there was no difference between groups in reported clarity of questions at Phase 2 (*p* = 0.843).

## Discussion

The current study builds upon previous research on supporting autistic people in employment interviews (e.g. Hillier et al., 2007; Kumazaki et al., 2019; Morgan et al., 2014; Scott et al., 2019; Smith et al., 2014, 2015; Strickland et al., 2013). The focus here, however, was on changes that *employers* can make to their questions, rather than the onus being entirely on the autistic person to adapt their interview technique. It is also, to our knowledge, the first study to compare how autistic adults perform against non-autistic adults during employment interviews; this is important given that, in most real-life job interview scenarios, autistic and non-autistic candidates will be competing with one another.

When standard (unadapted) interview questions were used in Phase 1, employment professionals provided lower ratings for both the quality of autistic interviewees’ answers and their overall impressions of them compared with non-autistic participants. Following adaptations to the questions, there was a significant improvement on both of these measures. While autistic participants were still rated less favourably than non-autistic participants on overall impressions in Phase 2, they showed a greater improvement in their answer quality than the non-autistic group, to the extent that differences between the groups were reduced in Phase 2. It is worth noting that there remained a marginally significant difference between groups with a medium effect size, which warrants further detailed examination. Employers’ ratings of their overall impressions of interviewees tentatively suggest that this disparity may relate to autistic interviewees being perceived as having poorer communication skills and appearing less confident. Overall, these results highlight the potential utility of combined interventions that take a two-pronged approach by focusing on training for both interviewees and interviewers (see Scott et al., 2019). They also demonstrate the effectiveness of relatively simple adaptations to questioning in facilitating the job interview performance of autistic adults, which also improve the quality of non-autistic interviewees’ responses.

As suggested by Lorenz et al. (2016), the social and cognitive demands of the typical interview process may present a major barrier for autistic people to successfully secure employment, placing them at a relative disadvantage compared with non-autistic individuals. Standard employment interviews often contain questions that have little explicit structure, particularly those regarding goals, aspirations, self-descriptions, and self-evaluations. Such questions are sufficiently ambiguous to allow most non-autistic candidates to employ IM tactics and present their skills, experience, and personal characteristics in a favourable manner while simultaneously avoiding revealing weaknesses (Campion et al., 1997). The current findings indicate that autistic candidates, however, find it difficult to interpret these sorts of questions, hindering their ability to formulate and recall a relevant and appropriately detailed response that conveys their best attributes and most relevant experience. Indeed, employer feedback indicated that autistic interviewees’ responses would be improved if they made fewer negative comments about themselves, highlighting reduced use of spontaneous IM tactics in this group. This may be underpinned by social cognitive differences that impact autistic interviewees’ ability to accurately gauge how they are perceived (e.g. Grainger et al., 2016; Sasson et al., 2018), or a reduced motivation to freely employ such tactics (Chevallier et al., 2012, but see Jaswal & Akhtar, 2018).

The present study developed novel adaptations to questions to support autistic difficulties and differences in IM (see Chevallier et al., 2012), self-monitoring (e.g. Grainger et al., 2016; Sasson et al., 2018), social cognition (see White, 2013), communication (e.g. Barnes & Baron-Cohen, 2012), memory (see Gaigg & Bowler, 2018), and executive functions such as generating, planning, and monitoring responses (Demetriou et al., 2018). One of the most salient adaptations to interview questions was the explicit and structured request for specific details, examples, and certain types of information, reducing the need for the interviewee to infer this implicitly (see White, 2013). This enabled interviewees to provide better quality answers, in line with findings from other areas of research showing that the accuracy and detail of autistic individuals’ eyewitness testimonies can be improved through more cued and structured questioning (see Maras, in press) while also diminishing differences between autistic and non-autistic candidates’ inclination and ability to employ IM tactics.

It may be critical that adaptations were made not only to the questions themselves but also to the way they were asked. Because autistic individuals often experience difficulties in executive function together with language issues that are more apparent when processing longer and more complex sentences (e.g. Riches et al., 2010), the interviewer asked each part of the question in turn, requiring a response from the interviewee before moving to the next part of the question. This added more structure to the question–answer process and facilitated responses that better demonstrated autistic interviewees’ personal skills and attributes. Interviewees were also provided with a printout of the questions, which reduced the need to hold multiple questions in mind or to infer what they might be asked next. Feedback from interviewees indicated that this was helpful, as evidenced by the reduced reports of cognitive difficulty in Phase 2 for the autistic group, alongside the very positive reports from both groups regarding how the printout helped to increase focus, reduce distraction, and provide reassurance about the progression of the interview.

Adaptations also improved employers’ overall impressions of both autistic and non-autistic interviewees in terms of their conscientiousness, communication skills, likeability, and ease to work with. Nevertheless, autistic interviewees were still rated more negatively than non-autistic interviewees (across both phases) in terms of their overall confidence, communication skills, likeability, and perceived ease to work with. This is somewhat surprising given that employers rated transcripts, rather than videos, of interviewees: Sasson et al. (2017) found that unfavourable impressions of autistic individuals diminished when their impressions were based solely upon conversational content without audio-visual cues. However, it may be significant that the raters in Sasson et al.’s study made judgements based on ‘thin slices’ of social behaviour, with transcripts featuring only 60 s of speech content. In contrast, interviews in the current study were between 5 and 30 min each. This may have increased the amount of information available for employers to base more negative perceptions of autistic participants on, and warrants further investigation. Autistic participants’ lower levels of employment experience (and possibly, therefore, reduced experience of interviews) may also have influenced this effect.

It may also be pertinent that employers in the present study were not informed of interviewees’ diagnoses. Previous research shows that disclosing one’s autism diagnosis can have a profound effect in improving others’ perceptions of them both in everyday contexts (e.g. Brosnan & Mills, 2016; Morrison et al., 2019; Sasson & Morrison, 2019) and in specific settings such as the Criminal Justice System (Crane et al., 2018; Maras, Crane, Walker, & Memon, 2019; Maras, Marshall, & Sands, 2019). Future research should examine whether diagnostic disclosure has a similar impact upon employer perceptions and whether disclosure together with adaptations to questions is enough to diminish differences in employers’ overall impressions between autistic and non-autistic interviewees.

The current study has several limitations that are important to acknowledge. First, participants completed a mock interview scenario with no specific job description. Although this promoted parity across participants from a wide range of backgrounds and abilities (deemed important for this preliminary investigation of question types, given that autistic participants had less employment experience than non-autistic participants), it nevertheless limited ecological validity. In particular, since role-specific knowledge and confidence in one’s abilities to successfully perform within that particular role are important drivers of interview performance (see Huffcutt, 2011), participants’ performance (and potentially also group differences) may have been underestimated in the absence of a job description. Indeed, feedback from both autistic and non-autistic interviewees indicated that interviews lacked the pressure of a real interview and that a job description would be necessary to enable them to give more complete answers.

Second, and relatedly, the current findings are based upon employer perceptions of interviewees based solely on interview transcripts. Focussing only on the content of their responses was deemed important in this initial exploratory investigation to test the effects of adaptations in improving the *quality* of interviewees’ responses (i.e. their content) without the confounds of behavioural cues such as demeanour. However, this precludes a more holistic understanding of how autistic candidates are perceived in employment interviews, which is pertinent given that non-verbal behaviours are also known factors in perceived job interview performance (e.g. Barrick et al., 2009). The finding that autistic interviewees were perceived as less confident than their non-autistic counterparts even in the absence of observable non-verbal behavioural cues highlights a need for future research to build on this by examining perceptions of their job interview performance more holistically, including behavioural cues. That both groups self-reported higher confidence in their responses to adapted questions is, nevertheless, provisionally encouraging.

Third, interviewer feedback was limited to ensure consistency across participants, thus restricting the social dynamics of the interview and, arguably, ecological validity. Yet, previous research has shown that highly structured employment interviews are more reliable and valid than unstructured interviews as they control different biases, making the same job-related information salient to all interviewers and helping to ensure that applicants are rated consistently across interviewers (Levashina et al., 2014; McCarthy et al., 2010).

Finally, autistic adults experience higher rates of co-occurring emotional disorders such as anxiety and depression (Hollocks et al., 2019), which are known to negatively distort information processing and memory retrieval (see Mathews & MacLeod, 2005) and may thus potentially hinder their performance in a job interview. It is a limitation that the present preliminary study did not include assessments of anxiety or depression, as this may have shed further light on employers’ comments that autistic interviewees made too many negative comments about themselves. This is an important avenue for future research.

It is worth mentioning that participants in the current study also fed back that offering preparation time by providing the questions in advance of the interview would be another helpful adaptation (see also Norris et al., 2020). For the purposes of the present study, it was felt that this would reduce experimental control, making it difficult to disentangle whether the adaptations to the questions per se were effective. This is, however, another important question for future research as it is a straightforward and cost-effective adaptation that employers can easily implement. Indeed, there are many other ways in which employers can adapt their interview process, such as environmental modifications (a quiet room without fluorescent strip lighting, a 90° seating angle to reduce pressure for eye contact, etc.), offering a working interview (whereby the applicant performs the duties of the job), or indeed removing interviews altogether and relying on competency-based exercises instead (Wood & Payne, 1998). These suggestions require detailed empirical examination.

While further inquiry is required along a number of avenues such as those outlined above, there are also more immediate implications from the current preliminary study. Most notably, that interview questions should be more explicit and specific in requesting the information that is required from an interviewee, rather than utilising standard underspecified questions that rely on candidates ‘reading between the lines’ to respond desirably with the requisite information. Breaking questions down into their component parts and providing a printout of questions can also be beneficial – for both autistic and non-autistic candidates. The onus of employment interventions to date has been predominantly targeted at autistic people, rather than on changes that employers can make to better enable them (Scott et al., 2019). The present findings are in line with a move away from a medical, impairment-focussed model towards an approach that considers contextual influences and the interaction between an individual and their environment (Shakespeare, 2013). An important next step towards encouraging employers to use evidence-based changes, such as the adaptations to questions demonstrated here, is to shift the focus from a perceived need to remediate autistic ‘impairments’ towards a greater understanding of autistic differences, which often only become disabilities when the social environment is not appropriately modified to accommodate them (see Milton, 2012; Scott et al., 2019). A recently accumulating body of research suggests that treatment programmes focussing on improving societal attitudes and acceptance of autistic people is critical in this respect (e.g. Morrison et al., 2019; Sasson & Morrison, 2019).

In conclusion, the current study provides the first test of interview question adaptations that employers can make for autistic candidates, rather than placing the responsibility solely on the autistic interviewee. Importantly, the adaptations were also effective at improving the interview performance of non-autistic participants, embodying the principles of universal design (Ostroff, 2001). Findings therefore demonstrate successful and straightforward adaptations to questions that employers could implement to level the playing field for autistic candidates while also being of benefit to non-autistic candidates.

## Supplemental Material

sj-pdf-1-aut-10.1177_1362361320981319 – Supplemental material for Ameliorating the disadvantage for autistic job seekers: An initial evaluation of adapted employment interview questions Click here for additional data file. Supplemental material, sj-pdf-1-aut-10.1177_1362361320981319 for Ameliorating the disadvantage for autistic job seekers: An initial evaluation of adapted employment interview questions by Katie Maras, Jade Eloise Norris, Jemma Nicholson, Brett Heasman, Anna Remington and Laura Crane in Autism

## Notes

1. In the present study, we use ‘identity first’ language (e.g. ‘autistic person’) to respect the views of many autistic people and their families (e.g. Kenny et al., 2016). We also recognise the wider natural variation of neurodiversity; thus, we use the term ‘non-autistic’ to describe our participants who do not have a diagnosis of autism nor present with social interaction challenges.

2. The review by Huffcutt (2011) also highlighted previous experience as an important factor; however, this was excluded from the current research because autistic participants are less likely to have occupational experience (e.g. Hendricks, 2010; Knapp et al., 2009).

3. In Phase 1, one autistic participant was unable to answer one question. In Phase 2, four autistic and one non-autistic participants were unable to answer one question. Predominantly this was because participants could not think of an example/situation to refer to (e.g. in Phase 2 for the question asking for an example of a time the interviewee had made a mistake in a professional situation).

4. As there were two sets of interview schedules (each comprising seven different questions) counterbalanced between phases, it was not appropriate to include individual ratings to each of the seven questions in a multivariate analysis of variance. Instead, a single rating score was calculated by averaging across the seven questions in each phase.

5. Although interviewees could see the questions before they were asked by the interviewer, they were asked to answer each section within each question in turn. However, if the person had clearly already covered the next section of the question in their response, the interviewer did not ask it and instead moved onto the next section.
