# Supplementary material for: Ameliorating the disadvantage for autistic job seekers: An initial evaluation of adapted employment interview questions
Source: Autism. 2020 Dec 18;25(4):1060–75. doi: 10.1177/1362361320981319 (PMC8108109; doi:10.1177/1362361320981319)
Supplement: sj-pdf-1-aut-10.1177_1362361320981319 – Supplemental material for Ameliorating the disadvantage for autistic job seekers: An initial evaluation of adapted employment interview questions [file sj-pdf-1-aut-10.1177_1362361320981319.pdf]

APPENDIX A. Interviewee instructions and Phase 1 (unadapted) and Phase 2 (adapted) questions for Schedule A and Schedule B [Square brackets where modifications may be needed, e.g., if a participant did not have any work experience]

General instructions

Phase 1: This is a mock job interview, but there is no specific role in mind. Please do your best to answer as if this were a real job interview.

Phase 2: This is a mock job interview, but there is no specific role in mind. Please do your best to answer as if this were a real job interview, and don't worry about repeating things from last time or telling me things you think I already know about you.

In this interview I am going to ask you about 7 work-related topics. For each topic I will first let you know what I am going to ask about (you don't need to provide a response to this bit). Then I will ask you between 1-3 specific questions in relation to that. Here is a print-out of the questions. I'll let you know which question we are on each time so you can easily follow it on the print-out as well.

The print out is there just a reminder - I will ask each question before you should respond. Please take your time to think of responses to the questions.

Interview schedule A

Q1 Phase 1 (unadapted): Tell me a little bit about yourself.

Q1 Phase 2 (adapted): First, I'm going to ask you to give me a short introduction to yourself:

- What are your best personal characteristics?
- What are your educational qualifications?
- What work experience do you have?

Q2 Phase 1 (unadapted): What were your responsibilities in your last job [volunteering experience]?

Q2 Phase 2 (adapted): I'm going to ask you about your responsibilities in your most recent job [volunteering role]:

- What was your job title?
- What were your most important tasks?
- What did you enjoy about the role?

Q3 Phase 1 (unadapted): Do you work well as part of a team?

Q3 Phase 2 (adapted): I'm going to ask you to give me an example of a time you've worked in a team:

- What was your role in the team?
- How did you work with the other team members to solve problems?

Q4 Phase 1 (unadapted): Tell me about a time where you had to work with someone who was difficult to get along with – how did/would you handle it?

Q4 Phase 2 (adapted): Think about a time you've worked with someone who was difficult to get along with. Please tell me:

- What was the situation?
- What did *you* do to try to resolve the situation?

Q5 Phase 1 (unadapted): What is your biggest work or educational achievement?

Q5 Phase 2 (adapted): I'm going to ask you to tell me about your biggest work or educational achievement:

- What work or educational achievement are you most proud of?
- Why is that?

Q6 Phase 1 (unadapted): What have you learned from your mistakes?

Q6 Phase 2 (adapted): I'm going to ask you about what you've learned from your mistakes:

- Please give me an example of when you've made a mistake in a professional situation.
- How did you rectify the mistake?
- What did you learn from it?

Q7 Phase 1 (unadapted): Where do you see yourself professionally in 5 years?

Q7 Phase 2 (adapted): Finally, I want you to think about the next 5 years in terms of your career. Please tell me:

- What kind of role would you like to be in in 5 years' time?
- What department, company, or institution would you like to be working in?

Interview schedule B

Q1 Phase 1 (unadapted): Tell me about any work or volunteer [or academic] experience that you have had

Q1 Phase 2 (adapted): First, I'm going to ask you about your work [volunteer] experience:

- What roles have you held previously?
- What responsibilities have you held previously?

Q2 Phase 1 (unadapted): What are some of your strengths?

Q2 Phase 2 (adapted): I'm going to ask about your strengths:

- What do you consider to be your main strengths (things that you are good at)?
- How have you used these strengths at work [in education]?

Q3 Phase 1 (unadapted): What are some of your weaknesses?

Q3 Phase 2 (adapted): Now I am going to ask about your weaknesses:

- What things do you find difficult or challenging in the context of your work [education/ volunteering]?
- How do you try to manage these weaknesses at work [education]?

Q4 Phase 1 (unadapted): Are you good at problem solving?

Q4 Phase 2 (adapted): Think of an example of a time you've solved a problem at work [education]. Please tell me:

- First of all, what the problem was
- What did you do to resolve that problem?
- What was the final result?

Q5 Phase 1 (unadapted): What experience do you have of managing high workloads?

Q5 Phase 2 (adapted): Think of an example of when you've had lots of tasks to complete in a limited amount of time. Please tell me:

- What the situation was
- What management strategies did you use?
- Were these strategies effective?

Q6 Phase 1 (unadapted): Tell me about a time you've disagreed with a colleague – how did/would you handle it?

Q6 Phase 2 (adapted): Think about a time you've disagreed with a colleague. Please tell me:

- What the disagreement was about
- What *you* did to resolve it?

Q7 Phase 1 (unadapted): What [would] makes you happy in a job?

Q7 Phase 2 (adapted): Finally, please think about what you consider to be the most important factors that make you happy in a job. Please tell me:

- What type of role would you like to be in?
- Why?

APPENDIX B. Scoring criteria for individual responses to questions

Please rate the content of the interviewee's response for each question using the following criteria

- **1 – Very poor: Very limited response** (e.g., a single yes/no answer with little to no elaboration; completely off-topic without referring back to the question at all)
- **2 – Poor: Satisfied none-to-very little of the question** (e.g., highly off-topic; inappropriate content; negative content or references (e.g., about self, co-workers, peers, previous work, employers, teachers, school, etc.); limited or no conjecture regarding themselves; no evidence of experience or personal characteristics that are relevant for employment)
- **3 – Fair: Satisfied limited portion of the question** (e.g., incomplete response; some deviation off topic; limited examples and details to support response; mildly negative references (e.g., about self, co-workers, peers, previous work, employers, teachers, school, etc.); examples and details are somewhat negative and portray subject in a mildly unfavorable light; partially theorizes as to how they did/would respond if never in that situation; response reveals some ability/personal characteristic to apply self in that situation
- **4 – Good: Satisfied adequate portion of question** (e.g., complete response without deviation from topic; examples and details that are relevant to the question and employment; adequate detail and examples to support response; examples and details are positive and portray interviewee in a favorable light; reflects on a basic level as to how they did/would respond if never in that situation; describes situation with a positive outcome; response suggests that he /she demonstrates this behavior on a regular basis
- **5 – Excellent: Fully satisfied all aspects of the question and is relevant to employment** (e.g., examples and details are provided where appropriate are highly relevant to the question and to employment; examples and details are positive and portray interviewee in a highly favorable light; complete response without deviation from topic; reflects on an elaborate level as to how they did/would respond in that situation; describes situation with a positive outcome; complete response and (where applicable) includes description of the situation, the tasks related to the situation, the actions that the interviewee took to resolve the situation, and result of the situation; interviewee's response suggests that he / she might demonstrate leadership in this area and will have significant practical experience).
